# Metabolites in Longissimus Lumborum From Beef Cattle Differ in Fat Content

**DOI:** 10.1002/fsn3.71345

**Published:** 2025-12-15

**Authors:** Zhen Ma, Fanrong Cui, Lei Chen, Xiao Wang, Wenjie Yu, Hao Jiang, Zongsheng Zhao, Xiangmin Yan

**Affiliations:** ^1^ College of Animal Science and Technology Shihezi University Shihezi Xinjiang China; ^2^ Institute of Animal Husbandry Xinjiang Academy of Animal Husbandry Urumqi Xinjiang China; ^3^ Yili Vocational and Technical College Yili Xinjiang China; ^4^ College of Animal Sciences Jilin University Changchun Jilin China

**Keywords:** differentially abundant metabolites, longissimus lumborum, meat quality, texture profile analysis

## Abstract

Improving meat quality is an important goal in beef production. Therefore, a deeper understanding of the biochemical characteristics that drive muscle development and fat deposition and their effects on final quality is important in the fields of meat science and animal production. Recent research has shown that metabolites can be utilized as biological indicators for measuring meat quality. Therefore, the general meat quality, texture profile, and differentially abundant metabolites in longissimus lumborum from 10 Kazakh (with low fat content) cattle (KAZ) and 10 Xinjiang brown (with high fat content) cattle (BC) were analyzed. A total of 800 metabolites were detected, among which were 47 differentially abundant metabolites (DAMs), including 1 amine, 1 heterocyclic compound, 1 tryptamine choline pigment, 18 amino acid derivatives, 3 benzene and its substituted derivatives, 5 fatty acyls, 3 glycoproteins, 5 nucleotides and their metabolites, 5 organic acids and their derivatives, and 5 carbohydrates and their metabolites. The top 5 up‐ and downregulated DAMs between the two breeds included Arg‐Lys, Pro‐Glu‐Val, 4‐(aminomethyl) indole, N6‐(2‐hydroxyethyl) adenosine, methyldopa, and biliverdin, which are related to metabolic pathways such as arginine and proline metabolism, galactose metabolism, endocrine resistance, and glycerolipid metabolism, potentially affecting meat quality, including sensory acceptability. These results suggest that metabolites are potentially useful biological indicators for measuring meat quality and provide an important theoretical basis for improving beef flavor and taste.

## Graphical abstract

Kazakh (KAZ) and Xinjiang brown cattle (BC) differ in meat quality. Forty‐seven meat quality‐related differentially abundant metabolites (DAMs) are involved in amino acid/carbohydrate/lipid metabolism, membrane transport, etc. Positive/negative correlation exists between DAMs.

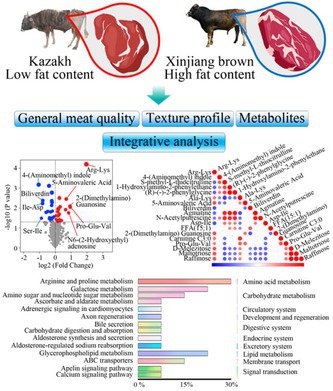

## 1 Introduction

By 2050, the global population is expected to surpass nine billion, with most people residing in urban areas. The demand for food is anticipated to increase, exceeding the current market demand by up to 70%. Therefore, the need for highly nutritious protein‐rich diets that include animal products is expected to increase. The global production of animal‐derived food is highly dependent on livestock, primarily ruminants, which are dominant in producing meat products adaptable to a wide range of ecosystems. Currently, ruminants are subjected to genetic selection and breeding to improve economically valuable traits and meet the specific requirements of various rearing conditions (Gaughan et al. 2019).

In many countries, beef is among the most in‐demand edible meats. The consumer preference for beef is generally attributed to flavor, tenderness, and/or a juicy texture (Kodani et al. 2017). Numerous genes regulate the formation of the meat traits, which are complex economic traits. Many economically relevant phenotypes in cattle differ among breeds or lines, indicating that the expression or determination of some traits depends on the genetic background. As a result, the characteristics of many breeds and lines are associated with their special performance in relation to a variety of traits, including fat content, carcass lean meat, muscle mass, growth rate and feed efficiency, reproduction parameters, and other traits, which are primary objectives of cattle breeding programs and the foundation for crossbreeding. These traits are considered the final phenotype or external properties because they are based on the theory of quantitative inheritance for the empirical interpretation of complex biological interactions and processes.

Meat has been analyzed via metabolomics, which can distinguish between distinct cow breeds and the geographical origin of beef (Zhang et al. 2021). In recent years, a new concept, “MEATabolomics,” which integrates meat metabolomics and muscle biology in domestic animals, has been proposed (Muroya et al. 2020). “Next‐generation phenotyping” may be caused by MEATabolomics, the former of which can improve the breeding value of prediction and selection strategies (Fontanesi 2016). In addition, high‐throughput omics technology has improved livestock production by contributing to the understanding of complex biological phenomena and disease resistance (Suravajhala et al. 2016). Therefore, metabolomics data may be used to improve the accuracy of genomic selection and the impact of breeding strategies on traits related to animal production by integrating multilevel high‐throughput omics approaches (Taniguchi et al. 2020).

Kazakh cattle (KAZ) is a native Chinese cattle breed primarily found in northern Xinjiang. In the past century, KAZ was used as the female parent, and purebred brown cattle (Swiss brown cattle, Alatau cattle, and Koström cattle) were used as sires to create a new variety to improve the meat production performance of Kazakh cattle. Xinjiang brown cattle (BC) appeared in the 1970s after hybridization. These meat‐type cattle were selected through hybridization to improve beef quality, especially meat with high fat deposition (Chen et al. 2022; Ma et al. 2023).

In this study, differentially abundant metabolites (DAMs) in the longissimus lumborum (LL) of KAZ and BC were analyzed via the LC‐QTRAP platform. Different compounds of various varieties were screened, and metabolic pathways associated with them were identified. Our research provides important theoretical information for improving beef cattle genetics and meat quality.

## 2 Materials and Methods

### 2.1 Animals

Ten 30‐month‐old native KAZ bulls with low fat content (554 ± 59 kg; slaughter rate: 55.23%) and 10 bc bulls with high fat content (605 ± 42 kg; slaughter rate: 55.41%) (Table S1) without hybridization of other sires were randomly selected from Xinjiang Tianlai Animal Husbandry Group Co. Ltd. (Bole, Xinjiang, China). These cattle were fasted for 12 h before being slaughtered. All the experimental cattle were fattened to slaughter under the same conditions according to the commercial standard (Table S2). The carcass was stored at 4°C for 72 h. All the meat samples were obtained from between the 12th and 13th right ribs of LL and stored at −20°C to determine the meat quality. At the same time, fresh tissue samples were cut into small 1 cm^3^ blocks and immediately stored in liquid nitrogen until further analysis. All procedures pertaining to the handling of experimental animals were conducted in accordance with the ARRIVE guidelines. All experiments were carried out in accordance with the approved guidelines of the Xinjiang Academy of Animal Husbandry (IACUC No. 2022006).

### 2.2 Analysis of the General Meat Quality Traits and Texture Profile

The total fat, total protein, and total water contents of LL were analyzed via near infrared reflectance (NIR) spectroscopy using a SupNIR‐1520 instrument, which was produced by Focused Photonics Inc. (Hangzhou, China).

The samples (4 cm × 4 cm × 4 cm) were placed in polyethylene plastic bags, heated in a water bath at 80°C to reach a core temperature of 70°C, cooled to room temperature under running water for 30 min, stored at 4°C overnight, wiped with filter paper to remove residual moisture, and reweighed to calculate the degree of cooking loss. The cooked samples were cut into six strips (1 cm × 1 cm × 2 cm) and eight strips (1 cm × 1 cm × 1 cm) parallel to the muscle‐fiber direction for shear force measurement and texture profile analysis (TPA), respectively. Shear force and TPA were estimated using a texture analyzer (TA.XT Plus, Stable Micro Systems Ltd., Godalming, UK). The TPA textural parameters were determined under the following conditions: crosshead speed, 1 mm/s; time interval between the first and the second compressions, 5.0 s; working distance, 75% strain; and trigger force, 5.0 g. From the resulting force–time curves, hardness, adhesiveness, springiness, cohesiveness, gumminess, chewiness, and resilience were determined.

To measure the rate of water loss, a modified Grau and Hamm method was applied. The rate of water loss was expressed as a percentage of the weight loss of 5 g meat samples immediately after being kept under a pressure of 2250 g for 5 min. Color parameters were immediately measured according to the Commission Internationale de L'Eclairage (CIE) L*, a*, and b* system (where L* indicates relative lightness, a* indicates relative redness, and b* indicates relative yellowness) using a MINOLTA CM 2002 spectrophotometer (illuminant: D65; visual angle: 10°; Minolta Camera Co., Osaka, Japan).

### 2.3 Liquid Chromatography–Mass Spectrometry (LC–MS)/MS Analysis

The samples were accurately weighed, and the metabolites were extracted using a 400 μL methanol: water (4:1, v/v) solution. The mixture was allowed to settle at −10°C and treated with a high‐throughput tissue crusher (Wonbio‐96c; Shanghai Wanbo Biotechnology Co. Ltd.) at 50 Hz for 6 min, followed by vortexing for 30 s and ultrasonication at 40 kHz for 30 min at 5°C. The samples were placed at −20°C for 30 min for precipitation. After centrifugation at 13,000×*g* at 4°C for 15 min, the supernatants were carefully transferred to sample vials for LC–MS/MS analysis.

The liquid chromatography–mass spectrometry (LC–MS) system for metabolomics analysis involved a Waters Acquity I‐Class PLUS ultrahigh‐performance liquid tandem Waters Xevo G2‐XS QT high‐resolution mass spectrometer. The column used was a Waters Acquity UPLC HSS T3 column. In positive ion mode, mobile phase A was an aqueous 0.1% formic acid solution, and mobile phase B was 0.1% formic acid in acetonitrile. In negative ion mode, mobile phase A was a 0.1% formic acid aqueous solution, and mobile phase B was 0.1% formic acid in acetonitrile. The injection volume was 1 μL.

A Waters Xevo G2‐XS QTOF high‐resolution mass spectrometer was used to acquire primary and secondary mass spectral data in MSe mode in the control of acquisition software (MassLynx V4.2, Waters). In each data acquisition cycle, dual‐channel data acquisition can be performed on both low and high collision energies simultaneously. For MS, the low collision energy was 2 V, the high collision energy range was 10–40 V, and the scanning frequency was 0.2 s. The parameters of the ESI ion source were as follows: capillary voltage, 2000 V (positive ion mode) and −1500 V (negative ion mode); cone voltage, 30 V; ion source temperature, 150°C; desolvation gas temperature, 500°C; backflush gas flow rate, 50 L/h; and desolvation gas flow rate, 800 L/h.

### 2.4 Data Preprocessing, Annotation, and Analysis

Nontarget metabolomics experiments and metabolome data statistics were performed on a platform at Biomarker Technologies (Beijing, China). The raw data collected using MassLynx V4.2 were processed by Progenesis QI software for peak extraction, peak alignment, and other data processing operations on the basis of the Progenesis QI software online METLIN database and Biomark's self‐built library for identification, and the theoretical fragment identification and mass deviation were within 100 ppm.

After the original peak area information was normalized to the total peak area, follow‐up analysis was performed. Principal component analysis and Spearman correlation analysis were used to evaluate the repeatability of the samples within groups and the quality control samples. The identified compounds were searched for classification and pathway information in the KEGG, HMDB, and lipidmaps databases. In accordance with the grouping information, the difference multiples were calculated and compared, and a t test was used to calculate the difference significance *p* value of each compound. The R language package ropls was used to perform OPLS‐DA modeling, and 200 permutation tests were performed to verify the reliability of the model. The VIP value of the model was calculated using multiple cross‐validations. The method of combining the difference multiple, *p* value, and VIP value of the OPLS‐DA model was adopted to screen the differentially abundant metabolites. The screening criteria were a FC > 2, a *p* value < 0.05, and a VIP > 1. The metabolites with differential KEGG pathway enrichment significance were calculated using a hypergeometric distribution test.

### 2.5 Statistical Analysis

The effects of different breeds on meat quality traits and texture profiles were analyzed using one‐way ANOVA, with different breeds as a fixed effect and replication as a random effect. The data are presented as the mean ± standard deviation (SD). Statistical analysis was performed via SPSS software (version 21.0; IBM, Chicago, IL, USA). *p* < 0.05 and *p* < 0.01 were considered to indicate statistical significance.

## 3 Results

### 3.1 Differences in Meat Quality Between KAZ and BC

As shown in Table 1, the slaughter weights of KAZ and BC were 554 ± 59 kg and 605 ± 42 kg (*p* < 0.05), respectively, whereas the slaughter rates were 55.23% ± 3.21% and 55.41% ± 4.70% (*p* > 0.05), respectively. In addition, the total water content did not differ between KAZ and BC (*p* > 0.05). However, KAZ and BC significantly differed in terms of meat quality traits, including lightness, redness, yellowness, pH, water‐holding capacity (WHC), cooking loss, shear force, total fat, and total protein (*p* < 0.05 or *p* < 0.01). In addition, compared with BC, KAZ had lower hardness, adhesiveness, cohesiveness, gumminess, and chewiness scores (*p* < 0.01). However, no differences were detected in terms of springiness and resilience (*p* > 0.05).

**TABLE 1:** Comparison of beef traits between Kazakh (KAZ) and Xinjiang brown cattle (BC).

General meat quality traits			Texture profile analysis		
	KAZ	BC		KAZ	BC
SLW (kg)	554 ± 59^a^	605 ± 42^b^	Hardness	9845.73 ± 3421.13^A^	24896.70 ± 4839.73^B^
SLR (%)	55.23 ± 3.21	55.41 ± 4.70	Adhesiveness	2.80 ± 1.13^A^	5.28 ± 2.42^B^
Lightness	29.46 ± 3.20^A^	33.98 ± 2.14^B^	Springiness	0.50 ± 0.03	0.51 ± 0.07
Redness	15.81 ± 1.^07A^	14.21 ± 1.22^B^	Cohesiveness	0.43 ± 0.04^A^	0.54 ± 0.05^B^
Yellowness	5.09 ± 1.19^a^	6.09 ± 0.87^b^	Gumminess	4069.66 ± 1136.66^A^	13217.56 ± 1614.29^B^
pH	6.28 ± 0.26^a^	6.04 ± 0.19^b^	Chewiness	2439.13 ± 806.66^A^	6906.37 ± 1716.73^B^
WHC	70.32 ± 3.83^a^	65.39 ± 4.23^b^	Resilience	0.18 ± 0.03	0.21 ± 0.04
Cooking loss	24.43 ± 0.96^a^	26.18 ± 1.74^b^			
Shear force	6.50 ± 0.85^a^	5.72 ± 0.50^b^			
Total fat	1.81 ± 0.50^A^	2.72 ± 0.41^B^			
Total protein	22.49 ± 0.73^a^	23.42 ± 0.93^a^			
Total water	73.24 ± 1.23	74.02 ± 0.84			

*Note:* Values with different lowercase (*p* < 0.05) or capital (*p* < 0.01) superscript letters indicate highly significant differences.

Abbreviations: SLR, slaughter rate; SLW, slaughter weight.

### 3.2 Overview of Identified Metabolites

**FIGURE 1:**
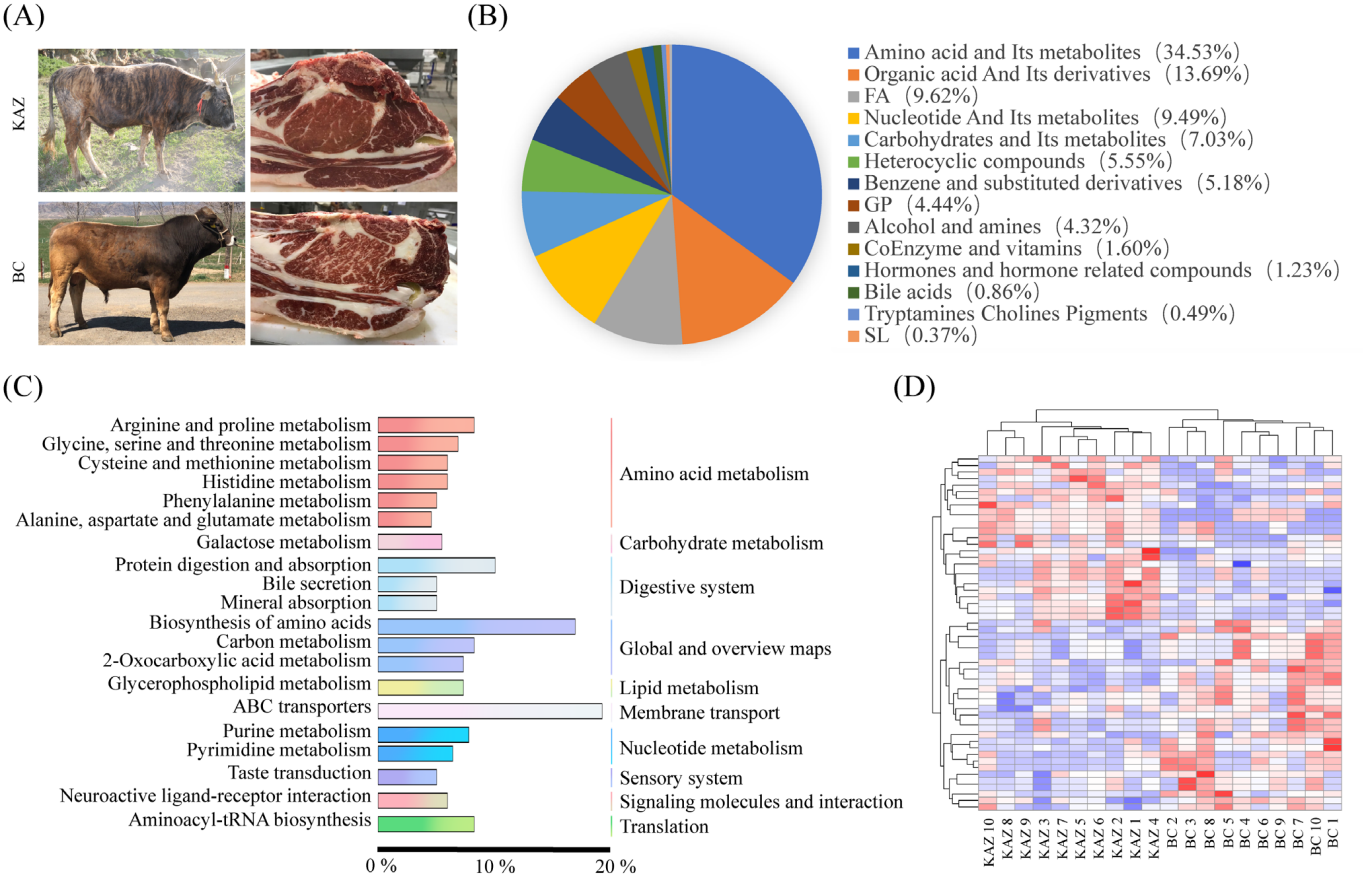
General information on the metabolomics analysis. (A) Representative images of KAZ and BC bulls and their rib eye muscles. (B) Classification of the detected metabolites. (C) Biological pathways associated with the detected metabolites. (D) Heatmap of DAM clustering.

A total of 800 metabolites, including 280 amino acids and their metabolites, 111 organic acids and their derivatives, 78 fatty acyls (FAs), 77 nucleotides and their metabolites, 57 carbohydrates and their metabolites, 45 heterocyclic compounds, 42 benzene and its substituted derivatives, 36 glycoproteins (GPs), 35 alcohols and amines, 13 coenzymes and vitamins, 10 hormones and hormone‐related compounds, 7 bile acids, 4 tryptamine choline pigments, three sphingolipids, and two other compounds, were detected in KAZ and BC at different marbling levels (Figure 1A,B, Table S3). All the detected metabolites were enriched in ABC transporters, biosynthesis of amino acids, arginine and proline metabolism, aminoacyl‐tRNA biosynthesis, carbon metabolism, and 163 other Kyoto Encyclopedia of Genes and Genomes (KEGG) pathways (Figure 1C, Table S4). Clustering analysis of the DAMs also revealed that all the individuals were clustered into the KAZ or BC categories (Figure 1D).

### 3.3 Identification of DAMs

After all 800 metabolites were identified, 47 DAMs were screened and identified according to the fold change and variable importance in the projection (VIP) value change (FC > 2, *p* value < 0.05, and VIP > 1; Table S5). The top 10 up‐ and downregulated DAMs, including Arg‐Lys, Pro‐Glu‐Val, 4‐(aminomethyl)indole, N6‐(2‐hydroxyethyl) adenosine, methyldopa, and biliverdin, among others, are displayed in Table 2.

**TABLE 2:** Top 20 differential metabolites (KAZ vs. BC).

Metabolites	Class	Fold change	*p*	VIP
Arg‐Lys	Amino acid and its metabolites	15.15	< 0.01	3.31
Pro‐Glu‐Val	Amino acid and its metabolites	3.70	< 0.01	2.25
2‐(Dimethylamino) Guanosine	Nucleotide and its metabolites	3.63	< 0.01	2.39
N6‐(2‐Hydroxyethyl) adenosine	Nucleotide and its metabolites	3.21	0.02	2.14
5‐Aminovaleric Acid	Amino acid and its metabolites	2.61	< 0.01	2.48
Guanidineacetic Acid	Organic acid and its derivatives	2.46	0.01	2.28
2‐Methylguanosine	Nucleotide and its metabolites	1.75	0.04	1.80
Carnitine C3:0	FA	1.53	< 0.01	2.40
D‐Ribono‐1,4‐lactone	Carbohydrates and its metabolites	1.44	0.04	1.95
Ala‐Lys	Amino acid and its metabolites	1.44	< 0.01	2.55
Carnitine C13:0	FA	0.67	0.04	1.86
Carnitine C9:1	FA	0.61	0.03	2.01
FFA (16:1)	FA	0.59	0.03	2.01
S‐methyl‐L‐thiocitrulline	Amino acid and its metabolites	0.58	< 0.01	2.85
Methyldopa	Amino acid and its metabolites	0.52	0.01	2.16
LPC (20:2/0:0)	GP	0.45	0.02	2.31
Biliverdin	Tryptamines cholines pigments	0.33	< 0.01	2.48
Asp‐Ile/Ile‐Asp	Amino acid and its metabolites	0.33	< 0.01	2.47
Ser‐Ile/Ser‐Leu	Amino acid and its metabolites	0.30	0.03	1.99
4‐(Aminomethyl) indole	Heterocyclic compounds	0.23	< 0.01	2.93

### 3.4 Exploring Potential Meat Quality‐Related DAMs and Pathways Between KAZ and BC

**FIGURE 2:**
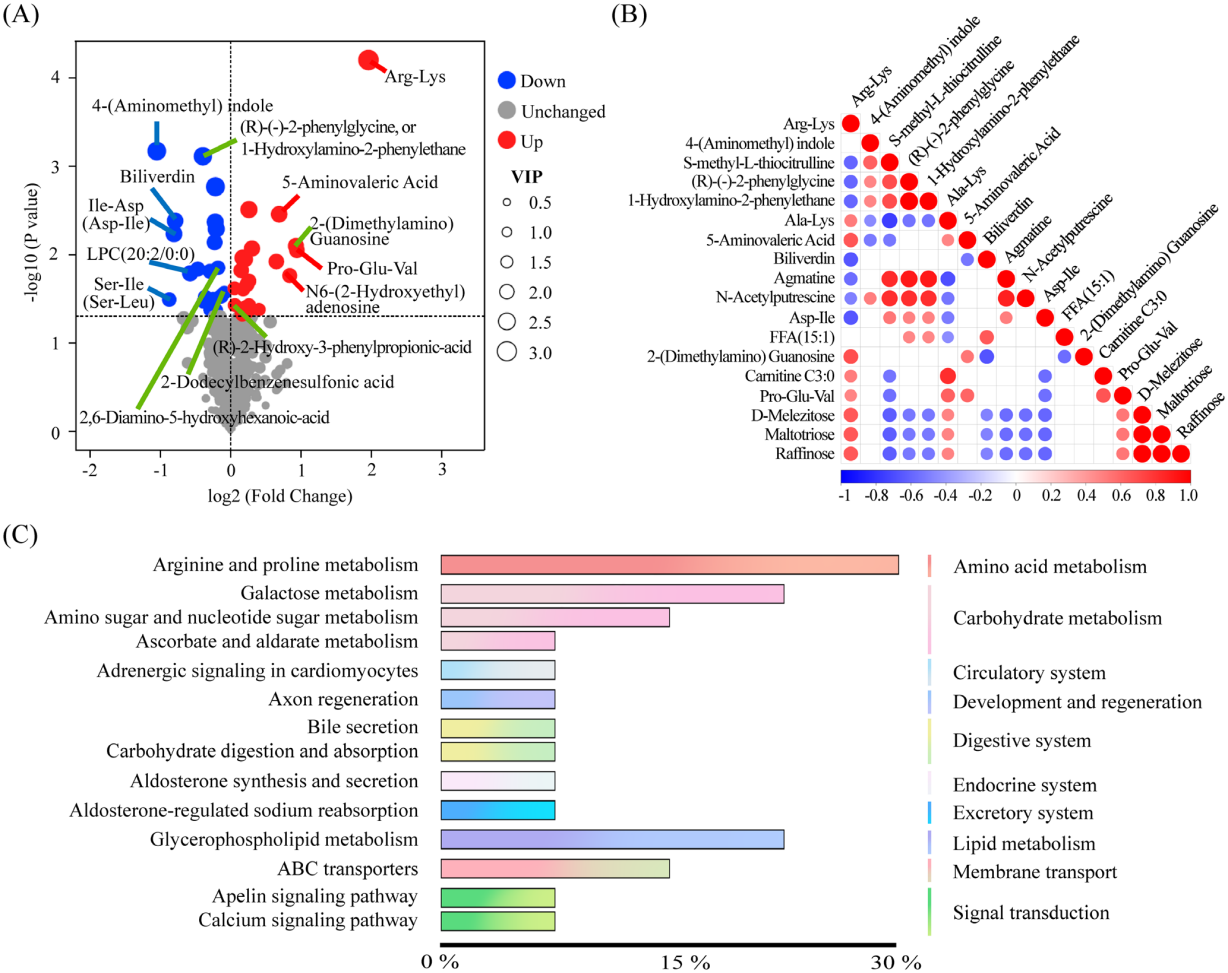
Meat quality‐related DAMs and their related biological pathways. (A) The volcano map shows the explored DAMs on the basis of their content, VIP (indicated by the red and blue lines), and ROC calculations (indicated by the green line). (B) Correlations between different DAMs. Only significant correlations are listed. The highest correlation value was 1, indicating a complete positive correlation (red), whereas the lowest correlation value was −1, indicating a complete negative correlation (blue). The color indicates a positive or negative correlation, which is more pronounced. The regions without color indicate that the calculated correlation is significantly lower than the significance threshold. (C) The first and second biological pathways associated with DAMs.

The volcano plot displays the most important DAMs between the two groups. Combining the area under the curve (AUC) and receiver operator curve (ROC) analysis results, (R)‐(−)‐2‐phenylglycine, 1‐hydroxylamino‐2‐phenylethane, (R)‐2‐hydroxy‐3‐phenylpropionic‐acid, 2,6‐diamino‐5‐hydroxyhexanoic‐acid, and 2‐(dimethylamino) guanosine were separated significantly between KAZ and BC (Figure 2A, Table S6). There were correlations among the DAMs. For example, raffinose was significantly positively correlated with maltotriose, D‐melezitose, Pro‐Glu‐Val, and Ala‐Lys but negatively correlated with S‐methyl‐L‐thiocitrulline, (R)‐(−)‐2‐phenylglycine, 1‐hydroxylamino‐2‐phenylethane, biliverdin, agmatine, N‐acetylputrescine, and Asp‐Ile (Figure 2B).

The top 10 biological pathways (first class) in which the DAMs were involved were amino acid metabolism, carbohydrate metabolism, the circulatory system, development and regeneration, the digestive system, the endocrine system, the excretory system, lipid metabolism, membrane transport, and signal transduction. The pathways (second class) in which the DAMs were involved were arginine and proline metabolism, galactose metabolism, amino sugar and nucleotide sugar metabolism, ascorbate and aldarate metabolism, adrenergic signaling in cardiomyocytes, axon regeneration, bile secretion, carbohydrate digestion and absorption, aldosterone synthesis and secretion, aldosterone‐regulated sodium reabsorption, glycerophospholipid metabolism, ABC transporters, the apelin signaling pathway, and the calcium signaling pathway (Figure 2C, Table S7).

## 4 Discussion

In this study, 800 metabolites were identified, among which many amino acids, fatty acyls, glycerophospholipids, carbohydrates, organic acids, nucleotides, and their metabolites or derivatives significantly differed between the two breeds. The metabolome data of KAZ and BC differed, potentially because of genetic factors. These findings were in accordance with those of previous research indicating that the amino acid composition of animal meat contributes to the scent and flavor of the meat and that the characteristics of the meat are breed specific (Dou et al. 2022; Zhan et al. 2022). Analysis of the general meat quality traits and texture profile revealed differences in meat color, pH, WHC, and tenderness. Intramuscular fat intake, lipid oxidation, metabolites, and pH are important factors that influence the color of meat. Therefore, for the LL, potential differences in the protein and fat compositions may exist between KAZ and BC during the improvement process, and the corresponding metabolites may also be significantly different. Carnitine (C13:0), carnitine (C9:1), FFA (16:1), S‐methyl‐L‐thiocitrulline, methyldopa, LPC (20:2/0:0), biliverdin, Asp‐Ile/Ile‐Asp, Ser‐Ile/Ser‐Leu, and 4‐(aminomethyl)indole may contribute to high fat deposition in BC cattle.

Previous studies have shown that corn is usually provided as a high‐energy diet and is the primary nutritional driver of intramuscular fat deposition. Dietary strategies to increase marbling in beef cattle primarily involve feeding high‐energy, grain‐based diets to cattle, as this leads to increased blood glucose levels that promote fat deposition. Adequate protein (e.g., soybean meal or alfalfa) has also been suggested to be required to support the metabolic processes involved in marbling without compromising lean yield. However, some studies have suggested that timed protein restriction during the finishing period could help increase marbling. Therefore, we comprehensively considered factors such as cost and management and used feed formulas such as those in Table S2. However, between KAZ and BC, significant differences in the growth performance, meat quality, and metabolites of the longissimus lumborum were found. We believe that the main reason for this is genetic factors.

Cattle breed is a primary factor driving variation in feed efficiency. Breeds showed consistent differences in average daily gain and body condition score, indicating that they have different abilities to utilize forage‐based energy and protein. This may be because these different breeds have inherent differences in intake, body composition, protein turnover, rumen fermentation, and methane production. These factors collectively determine how efficiently each breed converts dietary protein and energy into body weight gain (Andresen et al. 2020; Cantalapiedra‐Hijar et al. 2018). After nearly a century of crossbreeding, BC cattle formed a single population with stable genetic performance. The genetic structure, genetic diversity, and selection signatures differed greatly between KAZ and BC. BC is more representative of the beef cattle, in line with their breeding history, which involves crossbreeding (Wang et al. 2023). In this study, the slaughter weights of the KAZ and BC differed when they were fed the same finishing diet, reflecting profound genetic divergence in the utilization of dietary energy and protein for lean vs. fat deposition. In addition, different breeds of cattle exhibited distinct metabolomic profiles. Their metabolic responses to a feed efficiency challenge also differed, indicating that fundamental breed‐specific protein and energy metabolism pathways may differ (Yang et al. 2023).

In addition to flavor and meat color, meat tenderness is considered the most crucial sensory characteristic influencing customer satisfaction (Kodani et al. 2017). Metabolic pathways, including arginine and proline metabolism, galactose metabolism, endocrine resistance, and glycerolipid metabolism, play potential roles. The arginine, proline, and galactose metabolism pathways are directly related to sensory characteristics. Although citrulline‐to‐arginine production primarily occurs in the kidney, citrulline is also readily converted into arginine in other cell types, including adipocytes and myocytes (Wu et al. 2009). Our study revealed that the pH of the LL of KAZ was significantly greater than that of BC. The meat pH is associated with meat tenderness because of its influence on proteolytic activity (Jeleníková et al. 2008). Changes in pH are likely caused by increased mitochondrial levels, increased levels of tricarboxylic metabolites, and decreased levels of glycogen degradation enzymes and glycolytic metabolites. The intermediates of the TCA cycle and glycolysis are important biological markers of meat tenderness. Moreover, the key substances associated with these pathways, such as malic acid, glucose, and glucose‐6‐phosphate, also play important roles in meat tenderness. Similarly, carnosine and choline are indicators of tenderness. These findings indicated that the LL of KAZ resulted in a lower rate of degradation of high‐ and low‐molecular‐weight myofibrillar proteins than that of BC did, possibly due to differences in the levels of stimulated μ‐calpain and cathepsin B (Lomiwes et al. 2014). Our study also revealed that the Arg‐Lys level in the LL of KAZ was 15.15 times greater than that in the LL of BC. Arginine and lysine are generally believed to contribute to muscle fiber growth and the formation of more muscle, which may explain the greater WHC and shear force in the LL of KAZ (Zampiga et al. 2018; Zhang et al. 2020). Moreover, these findings indicated that, as a local variety, the adaptability of KAZ occurred over a long period, and the high levels of muscle‐related amino acids indicate its strong nutrient utilization and environmental adaptability. However, this may also lead to a poorer taste. In the process of improving from KAZ to BC, muscle growth and muscle fiber composition were strongly related, whereas fat content increased without reducing meat production. This difference is also related to the breeding of the varieties and the feeding methods used, ranging from grazing to farming.

Early after the death of animals, amino acids are the main energy source in muscles because they can be degraded as a component of the TCA cycle or as intermediate compounds that can be converted into a component of the TCA cycle. These findings indicate that arginine and proline metabolism, glycine, serine and threonine metabolism, cysteine and methionine metabolism, histidine metabolism, and alanine, aspartate and glutamate metabolism play very important roles in beef tenderness by affecting the TCA cycle. In addition, our study revealed significant changes in galactose metabolism, endocrine resistance, and glycolipid metabolism in the LLs of KAZ and BC, which may synergistically affect the tenderness of beef via amino acid metabolism (Shi et al. 2022). Creatine is also important for muscle quality because it affects protein synthesis by increasing the access of myosin to ATP. Guanidineacetic acid (GAA), also called guanidinoacetate or glycocyamine, is the precursor to creatine. Therefore, changes in the composition of amino acids and their metabolic pathways, especially those related to energy metabolism, may be important reasons for the differences in shear force, hardness, adhesiveness, springiness, cohesiveness, and chewiness in LL, thereby leading to greater tenderness in BC.

Although meat color is not a reliable indicator of meat quality, many consumers reference it before purchasing (Liu et al. 2022). Intramuscular fat intake, lipid oxidation, metabolites, and pH are important factors that influence the color of meat (Mancini and Hunt 2005). Our study revealed that the lightness and yellowness of the LL of BC were significantly greater than those of the LL of KAZ, whereas the redness was significantly lower than that of KAZ, indicating that the LL of KAZ contains more muscle fibers, whereas that of BC has a greater intrinsic fat content. Compared with that of the BC muscle, the pH of the LL of KAZ was greater. This may also be an important reason for the lower lightness and yellowness but greater redness in the LL of KAZ, which are related to differences in myoglobin and myofibril modifications. In addition, our study revealed that more than 9% of FAs are present in the DAMs of the LL between KAZ and BC. Interestingly, some studies have shown that changing the fatty acid profile does not change the color of meat. The difference in meat color between KAZ and BC may be due mainly to changes in fat content and the degree of fatty acid oxidation, eventually leading to the oxidation of myoglobin and discoloration of the meat (Domínguez et al. 2019).

In addition to meat color, meat flavor is considered the most crucial sensory characteristic influencing customer satisfaction. Our study revealed that many DAMs are involved in galactose metabolism, carbohydrate digestion, and absorption. Carbohydrates contribute in one way or another to the final flavor of beef (Aaslyng and Meinert 2017). These findings suggested that the flavors of KAZ and BC might differ because of differences in carbohydrate metabolism (Umbayev et al. 2020). In addition, the content and composition of lipids can strongly affect the flavor of beef. Our study revealed that the DAMs between KAZ and BC were FAs. The DAMs of KAZ and BC include many FAs, such as carnitine C3:0, carnitine C13:0, and carnitine C9:1, and GPs, such as LPC (16:0/0:0), LPC (20:2/0:0), and SL; however, many DAMs are enriched in pathways such as glyceroptosis metabolism, indicating that changes in these lipid metabolites and pathways may lead to changes in lipid levels and regulate the intramuscular fat content and composition, which in turn affects the flavor of beef (Kerth and Miller 2015). Moreover, the flavor of beef is strongly influenced by the protein level and its amino acid composition. Our study revealed that the top 20 DAMs in the LLs of KAZ and BC contained many amino acids and their metabolites, including Arg‐Lys, Pro‐Glu‐Val, and 5‐aminovaleric acid. Many DAMs are enriched in amino acid metabolism pathways, such as arginine and proline metabolism and glycine, serine, and threonine metabolism pathways, indicating that the differences and metabolic changes in these amino acids may affect the protein composition and content of beef, thereby leading to differences in the flavor of the LL between KAZ and BC.

Finally, we found positive/negative correlations between the DAMs. According to other researchers, oxidative stress, mitochondrial dysfunction, and depletion of metabolites that can regenerate NADH affect the color stability of aged beef, and therefore, considering the potential influence of the age at slaughter on the meat quality is important (Ramanathan et al. 2020). These findings indicate that metabolites, such as glucuronides, nucleotides, free amino acids, nucleosides, and carnitines, are involved in the response to meat packing and processing. Metabolites related to myoglobin and antioxidant activity are also strongly associated with meat quality (Subbaraj et al. 2016). Glutamate and 5′‐ribonucleotides, such as guanylate and inosinate, are the main substances that affect the umami of meat (Ninomiya 2016). Moreover, the nonvolatile constituents in fresh meat (such as organic acids, inorganic salts, amino acids, peptides, and sugars) and flavor enhancers (such as monosodium glutamate, guanosine 5′‐monophosphate, and inosine 5′‐monophosphate) are important for the basic taste of cooked meat (Khan et al. 2015). Researchers confirmed strong correlations between the amounts of odorants and phosphoric acid, lactic acid, decanoic acid, and glutamine (Ueda et al. 2021). Our results are also in accordance with the results that bitter‐tasting amino acids (such as leucine and isoleucine) and umami‐representative amino acids (such as alanine) are significantly present in different breeds of beef cattle (Lee et al. 2019). Owing to its fundamental role in initiating anabolic processes in muscle, including the synthesis of myofibrillar protein, leucine is associated with the isoleucine and valine biosynthesis pathways and can be affected by growth patterns between breeds. However, we also observed that valine, which is a bitter‐tasting‐related amino acid, is more prevalent in KAZ, indicating that the improvement process may not be able to improve all aspects of meat quality at once. In this study, KAZ and BC were fed under the same conditions, indicating that although different diets can effectively improve meat quality during the feeding process, the genetic basis of the variety is still a potentially important influencing factor.

## 5 Conclusion

Our study revealed that many DAMs were present in the LLs of KAZ and BC, which have different meat qualities. These metabolites affect the tenderness, color, and flavor of LL, thereby leading to differences in meat quality through arginine and proline metabolism; glycine, serine, and threonine metabolism; galactose metabolism; and glycerophospholipid metabolism. Through the amino acid and carbohydrate metabolism pathways, Arg‐Lys, Pro‐Glu‐Val, 2‐(dimethylamino) guanosine, N6‐(2‐hydroxyethyl) adenosine, 5‐aminovaleric acid, guanidineacetic acid, 2‐methylguanosine, carnitine (C3:0), D‐ribono‐1,4‐lactone, and Ala‐Lys contribute to muscle growth and muscle fiber composition in KAZ, whereas carnitine (C13:0), carnitine (C9:1), FFA (16:1), S‐methyl‐L‐thiocitrulline, methyldopa, LPC (20:2/0:0), biliverdin, Asp‐Ile/Ile‐Asp, Ser‐Ile/Ser‐Leu, and 4‐(aminomethyl) indole may contribute to high fat deposition in BC cattle through the lipid metabolism pathway. These results provide a theoretical basis for genetic improvement and meat quality trait selection in beef cattle.

## Author Contributions

**Zhen Ma:** investigation, conceptualization, validation, data curation, and writing – original draft preparation. **Fanrong Cui:** investigation and formal analysis. **Lei Chen:** investigation and formal analysis. **Xiao Wang:** validation and investigation. **Wenjie Yu:** writing – review and editing and visualization. **Hao Jiang:** supervision and visualization. **Zongsheng Zhao:** conceptualization; resources, supervision, and project administration. **Xiangmin Yan:** conceptualization, methodology, validation, data curation, supervision, project administration, funding acquisition, and writing – review and editing.

## Conflicts of Interest

The authors declare no conflicts of interest.

## Supporting information


**Data S1:** fsn371345‐sup‐0001‐Table.xlsx.

## Data Availability

The data that support the findings of this study are available on request from the corresponding author.
